# Feasibility of using abbreviated scan protocols with population-based input functions for accurate kinetic modeling of [^18^F]-FDG datasets from a long axial FOV PET scanner

**DOI:** 10.1007/s00259-022-05983-7

**Published:** 2022-10-04

**Authors:** Hasan Sari, Lars Eriksson, Clemens Mingels, Ian Alberts, Michael E. Casey, Ali Afshar-Oromieh, Maurizio Conti, Paul Cumming, Kuangyu Shi, Axel Rominger

**Affiliations:** 1 Advanced Clinical Imaging Technology, Siemens Healthcare AG, Lausanne, Switzerland; 2 Department of Nuclear Medicine, Inselspital, Bern University Hospital, University of Bern, Freiburgstrasse 18, 3010 Bern, Switzerland; 3 Siemens Medical Solutions USA, Inc., Knoxville, TN USA; 4 Department of Oncology and Pathology, Medical Radiation Physics, Karolinska Institutet, Stockholm, Sweden; 5 School of Psychology and Counselling, Queensland University of Technology, Brisbane, Australia

**Keywords:** LAFOV PET, Kinetic modeling, Parametric imaging, FDG

## Abstract

**Background:**

Accurate kinetic modeling of 18F-fluorodeoxyglucose ([^18^F]-FDG) positron emission tomography (PET) data requires accurate knowledge of the available tracer concentration in the plasma during the scan time, known as the arterial input function (AIF). The gold standard method to derive the AIF requires collection of serial arterial blood samples, but the introduction of long axial field of view (LAFOV) PET systems enables the use of non-invasive image-derived input functions (IDIFs) from large blood pools such as the aorta without any need for bed movement. However, such protocols require a prolonged dynamic PET acquisition, which is impractical in a busy clinical setting. Population-based input functions (PBIFs) have previously shown potential in accurate Patlak analysis of [^18^F]-FDG datasets and can enable the use of shortened dynamic imaging protocols. Here, we exploit the high sensitivity and temporal resolution of a LAFOV PET system and explore the use of PBIF with abbreviated protocols in [^18^F]-FDG total body kinetic modeling.

**Methods:**

Dynamic PET data were acquired in 24 oncological subjects for 65 min following the administration of [^18^F]-FDG. IDIFs were extracted from the descending thoracic aorta, and a PBIF was generated from 16 datasets. Five different scaled PBIFs (sPBIFs) were generated by scaling the PBIF with the AUC of IDIF curve tails using various portions of image data (35–65, 40–65, 45–65, 50–65, and 55–65 min post-injection). The sPBIFs were compared with the IDIFs using the AUCs and Patlak *K*_i_ estimates in tumor lesions and cerebral gray matter. Patlak plot start time (t*) was also varied to evaluate the performance of shorter acquisitions on the accuracy of Patlak *K*_i_ estimates. Patlak *K*_i_ estimates with IDIF and t* = 35 min were used as reference, and mean bias and precision (standard deviation of bias) were calculated to assess the relative performance of different sPBIFs. A comparison of parametric images generated using IDIF and sPBIFs was also performed.

**Results:**

There was no statistically significant difference between AUCs of the IDIF and sPBIFs (Wilcoxon test: *P* > 0.05). Excellent agreement was shown between Patlak *K*_i_ estimates obtained using sPBIF and IDIF. Using the sPBIF_55–65_ with the Patlak model, 20 min of PET data (i.e., 45 to 65 min post-injection) achieved < 15% precision error in *K*_i_ estimates in tumor lesions compared to the estimates with the IDIF. Parametric images reconstructed using the IDIF and sPBIFs with and without an abbreviated protocol were visually comparable. Using Patlak *K*_i_ generated with an IDIF and 30 min of PET data as reference, Patlak *K*_i_ images generated using sPBIF_55–65_ with 20 min of PET data (t* = 45 min) provided excellent image quality with structural similarity index measure > 0.99 and peak signal-to-noise ratio > 55 dB.

**Conclusion:**

We demonstrate the feasibility of performing accurate [^18^F]-FDG Patlak analysis using sPBIFs with only 20 min of PET data from a LAFOV PET scanner.

**Supplementary Information:**

The online version contains supplementary material available at 10.1007/s00259-022-05983-7.

## Introduction

With the recent technological developments in positron emission tomography (PET), kinetic modeling and parametric imaging of dynamic PET datasets have shown increased potential for improved disease diagnosis, therapeutic response monitoring, and drug development [1–4]. Physiologically based kinetic models often require an accurate knowledge of the time-dependent concentration of the PET tracer in the arterial blood, which is commonly known as the arterial input function (AIF). The current gold standard method to derive the AIF entails serial arterial blood sampling throughout the entire dynamic PET scan. Due to its invasiveness, arterial blood sampling is rarely applied outside of a research setting. Measurement of an image-derived input function (IDIF) is a non-invasive alternative, but often suffers from partial volume effects if a large vascular structure is not present in the field-of-view (FOV), as occurs in some neuroimaging studies [5]. Hence, IDIF extraction methods for head imaging usually require a co-registered high-resolution anatomical image (i.e., MRI) for the delineation of arteries and partial volume correction [6–8].

With the introduction of long axial FOV (LAFOV) PET/CT scanners, IDIFs can be derived from various large vascular structures or blood pools (i.e., aorta, left ventricle), minimizing the partial volume effects [9, 10]. Furthermore, the increased sensitivity of these systems enables the use of short frame durations in the reconstruction of early PET frames [11–13], allowing a more detailed capture of the IDIF curve peaks. Nevertheless, approximately hour-long dynamic [^18^F]-FDG PET acquisitions from the time of tracer administration are still required to capture the whole IDIF from the time of tracer administration, making these protocols cumbersome in a busy clinical setting. The development of alternative methods to enable dynamic imaging protocols which are compatible with routine clinical procedures is necessary, and recently developed high-sensitivity scanners with long axial FOV (LAFOV) may make this possible. Recent work with LAFOV PET systems has shown that abbreviated dynamic imaging protocols can be used to extract net tissue influx of [^18^F]-FDG, i.e., the Patlak slope, known as *K*_i_ (ml g^−1^ min^−1^) with a total scan duration of 10 min [14]. We have also previously shown that *K*_i_ and some of the kinetic microparameters can be estimated with low bias and good precision with a total scan duration of 15–20 min [15]. However, one major limitation of these protocols is that they require dual-time point scanning to capture the early and late parts of tracer dynamics, making these protocols more challenging to be applied in practice. Furthermore, a second CT scan for PET data corrections and registration of early and late scans are required for accurate parametric imaging using these protocols with resultant additional radiation dose.

As an alternative, population-based input functions (PBIFs) [16–18] are an attractive alternative for Patlak modeling of [^18^F]-FDG datasets [19–21] to derive kinetic macroparameters such as net tracer influx (*K*_i_) and tracer distribution volume (DV; ml g^*−*1^). In this study, we exploit the high sensitivity and temporal resolution of a LAFOV PET system to explore the use of PBIFs with abbreviated protocols in kinetic modeling of dynamic [^18^F]-FDG datasets. We investigate the effect of different scanning periods on the accuracy of PBIF scaling and systematically explore the performance of abbreviated protocols with PBIFs to obtain reliable kinetic parameters from [^18^F]-FDG datasets in a series of oncological patients.

## Materials and methods

This work includes [^18^F]-FDG PET data from a clinically heterogeneous group of 24 oncological subjects (9 females, 15 males; mean age: 60 ± 15 years, mean weight: 77 ± 17 kg). The dataset was randomly separated into a PBIF generation group (*n* = 16) and a validation group (*n* = 8). There were no statistically significant differences between the mean age, weight, and injected doses of the two groups (unpaired *t*-test, *p* > 0.05). The subjects were scanned as part of a dynamic imaging protocol, where dynamic PET emission data were acquired for 65 min using Biograph Vision Quadra (Siemens Healthineers) LAFOV PET/CT system. Intravenous bolus injection of [^18^F]-FDG (mean activity 235 ± 51 MBq) to the left or right arm was performed approximately 15 s after the start of the PET acquisition using a 150-cm-long extension line. Following the PET scan, a low-dose CT scan was used for anatomical information and PET data corrections. The list-mode PET data were reconstructed using 62 frames with the following frame durations: 2 × 10 s, 30 × 2 s, 4 × 10 s, 8 × 30 s, 4 × 60 s, 5 × 120 s, and 9 × 300 s. The initial two 10-s frames were employed to account for the time delay between the start of PET acquisition and [^18^F]-FDG administration. Image reconstruction was performed using the PSF + TOF reconstruction algorithm, with 4 iterations and 5 subsets with a voxel size of 1.65 × 1.65 × 1.65 mm^3^. A Gaussian filter with a 2 mm FWHM was used to smooth the images.

The descending thoracic aorta and whole brain volumes of interests (VOIs) were generated using a deep-learning-based method implemented in a research prototype software (MIWBAS version 1.0, Siemens Medical Solutions USA, Inc) [9, 22]. Brain gray matter VOIs were extracted utilizing a standard space [^18^F]-FDG healthy brain template (available in PMOD v.4.1, PMOD Technologies, Zurich, Switzerland). In addition, an experienced nuclear medicine physician manually delineated 34 tumor lesions from the 8 testing sets using an isocontour tool (PMOD 4.1, threshold set to 50% of max value).

The IDIFs were extracted using the VOI from the descending thoracic aorta, which was isotopically eroded by 6 mm in all directions to reduce partial volume and motion effects. The PBIF was derived using the 14 datasets in the PBIF generation group using the following steps: The IDIFs were normalized to their respective area under curves (AUC). Next, the normalized curves were fitted using Feng’s input function model [23], which is a sum of a gamma variate function with two exponentials with seven parameters (Eq. 1). The fitted curves were adjusted to population mean time delay. Then, the resulting curves were averaged to generate the PBIF

1 $${C}_{p}\left(t\right)={(A}_{1}t-{A}_{2}-{A}_{3}){e}^{-{\lambda }_{1}t}+{A}_{2}{e}^{-{\lambda }_{2}t}+{{A}_{3}e}^{-{\lambda }_{3}t}$$

where *λ*_1_, *λ*_2,_ and *λ*_3_ are the eigenvalues of the model and *A*_1_, *A*_2,_ and *A*_3_ are the coefficients of the model [23].

During the evaluation of the PBIF, five scaled PBIFs (sPBIFs) were generated by scaling the PBIF to the AUC of IDIF curves tails using various time periods (35–65 min, 40–65 min, 45–65 min, 50–65 min, and 55–65 min post-injection). Each of these sPBIFs was evaluated against the IDIFs by comparing AUCs and Patlak *K*_i_ estimates in tumor lesions and brain gray matter. Once the best performing timing window to generate sPBIF was determined, the Patlak analysis was repeated with this sPBIF with varying Patlak start time (t*) to evaluate the performance of Patlak analysis with a sPBIF with shortened PET acquisitions. Patlak fittings were performed utilizing the open-source COMKAT software package (Compartment Model Kinetic Analysis Tool, v.4.1) [24] using MATLAB (v2021, The MathWorks, Inc). Here, we first compare the estimated Patlak *K*_i_ values computed using IDIF and sPBIF at different t* values. Second, to assess the performance of abbreviated dynamic imaging protocols with a sPBIF to full protocols with an IDIF, mean bias and precision (standard deviation of bias) of Patlak *K*_i_ estimates with a sPBIF and varying t* values were calculated using *K*_i_ estimates obtained using an IDIF and t* = 35 min as reference.

Parametric Patlak *K*_i_ images are also reconstructed using the IDIF, the best performing sPBIF, and different PET data durations. Parametric images were reconstructed using the direct Patlak method implemented in a dedicated parametric imaging software prototype (Siemens Healthineers) which employs a nested expectation maximization algorithm [25]. Parametric images were reconstructed using the PSF + TOF method with 8 iterations and 5 subsets, 30 nested loops, and were smoothed using a 2-mm FWHM Gaussian filter [9]. Quantitative evaluation of images was performed by computing non-absolute and absolute relative change (% RC), the structural similarity index measure (SSIM), and peak signal-to-noise ratio (PSNR) relative to corresponding images obtained with the IDIF and t* = 35 min.

## Results

**Fig. 1:**
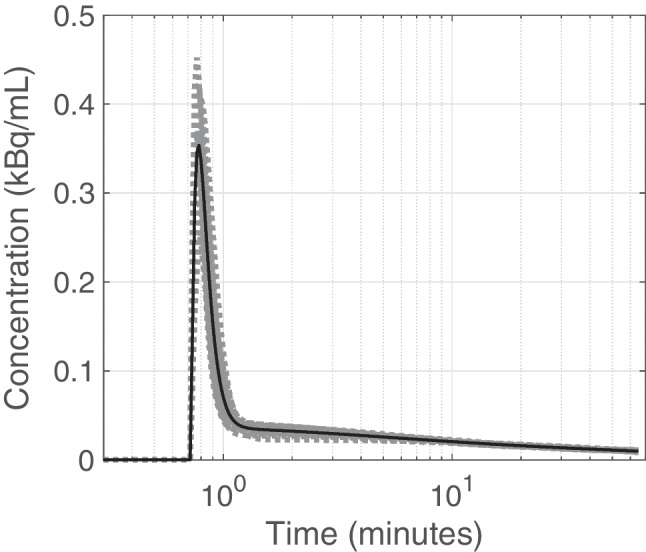
The computed PBIF (solid curve) and distribution of normalized IDIFs from 16 subjects (shaded gray area), presented as a semilog plot to accentuage agreement at the early phase. The *y*-axis represents the blood [^18^F]-FDG concentration of individual curves normalized to their respective AUCs

**Fig. 2:**
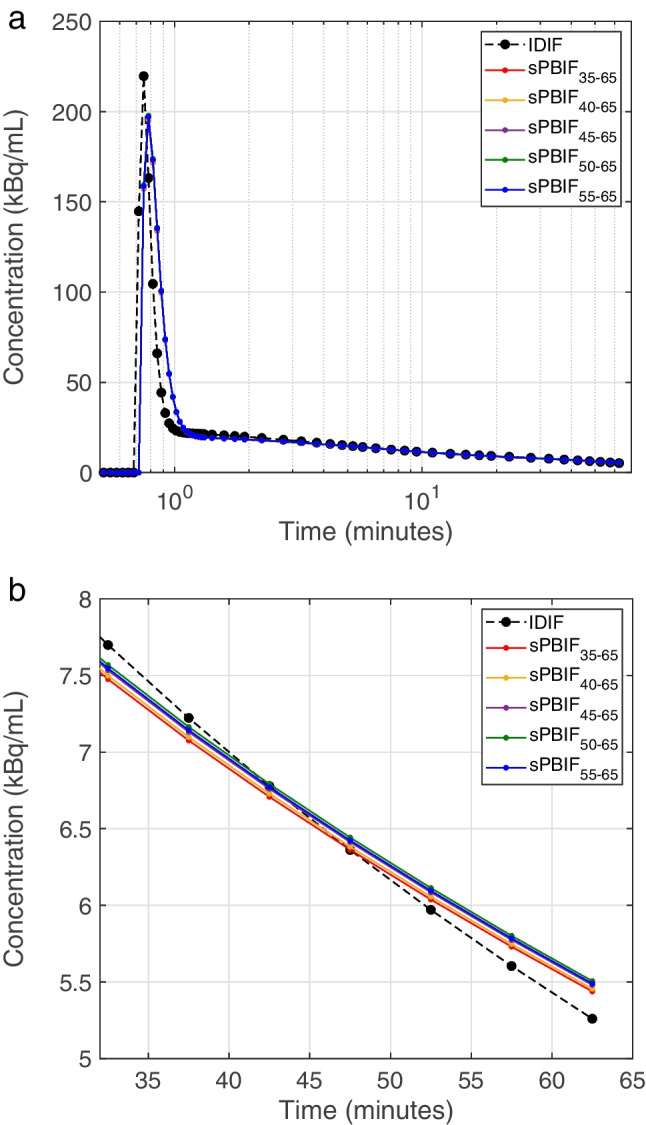
A representative IDIF plotted with sPBIFs scaled using image data from different scanning periods. The full input function curve (**a**) and tail of the curve (**b**) are illustrated separately

The distribution of AUC-normalized IDIFs from the training set and the generated PBIF curve are shown in Fig. 1. The estimated parameters from fitting the generated PBIF with the Feng’s model [4] were *τ* = 0.72 min, *A*_1_ = 15.9, *A*_2_ = 0.02, *A*_3_ = 0.02, *λ*_1_ = 17.8 min^−1^, *λ*_2_ = 0.18 min^−1^, and *λ*_3_ = 0.01 min^−1^. Figure 2 shows each of the sPBIFs plotted together with the IDIF from a representative subject in semi-logarithmic scale. This figure illustrates that the sPBIFs visually agreed well with the IDIF even though the true amplitudes of the peak were slightly underestimated. When computed for the eight validation datasets, the mean AUC (kBq min^−1^ ml^−1^) was 550 ± 54 for IDIF and 542 ± 45 for sPBIF_35–65_, 544 ± 45 for sPBIF_40–65,_ 547 ± 44 for _s_PBIF_45–65_, 550 ± 43 for sPBIF_50–65_, and 554 ± 43 for _s_PBIF_55–65_ (supplementary Fig. 1). There were no statistically significant differences among the AUCs (0–65 min) of sPBIFs and the IDIF.

Table 1 shows the *R*^2^, bias and precision of *K*_i_ values in tumor lesions and brain gray matter, calculated using the Patlak model (t* = 35 min) with different sPBIFs, compared against the corresponding *K*_i_ estimates obtained with the IDIF. As shown in Table 1 (part A), all five sPBIFs served to estimate the tumor lesion *K*_i_ with less than 4% bias and good precision (standard deviation of bias < 10%). Table 1 (part B) shows *R*^2^, the bias and precision of the error of *K*_i_ estimates in brain gray matter. The mean bias of the estimates calculated using each of the sPBIFs ranged from 1.9 to 4.3%. For both tumor lesions and brain gray matter, excellent agreement between Patlak *K*_i_ values estimated using each sPBIF and IDIF (*R*^2^ > 0.98). Although all of the sPBIFs showed similar performance with very good resemblance to the IDIFs, sPBIF_55–65_ yielded the lowest bias and standard deviation of bias in tumor and brain gray matter *K*_i_ values. Therefore, it can be said that the last 10 min of a 65-min long dynamic [^18^F]-FDG scan can be accurately used for scaling of a PBIF. We used sPBIF_55–65_ in the evaluation of the abbreviated protocols with varying Patlak start time (t*) values in the rest of this work.

**Table 1:** *R*-squared, comparison of bias, and precision (standard deviation of bias) of [^18^F]-FDG *K*_i_ estimates using PBIFs scaled with image data from different scan intervals compared against estimates with IDIF

	sPBIF_35–65_	sPBIF_40–65_	sPBIF_45–65_	sPBIF_50–65_	sPBIF_55–65_
A: Tumor lesions					
*R*^2^	0.998	0.999	0.999	0.999	0.999
Bias	4.0%	3.6%	3.0%	2.4%	1.5%
Precision	7.9%	7.8%	7.6%	7.3%	6.8%
B: Brain gray matter					
*R*^2^	0.981	0.982	0.984	0.986	0.989
Bias	4.3%	3.8%	3.3%	2.6%	1.9%
Precision	4.2%	4.1%	3.9%	3.7%	3.3%

Patlak linearization start time t* was set to 35 min post-injection. Results are shown for tumor lesions (A) and brain gray matter (B)

**Fig. 3:**
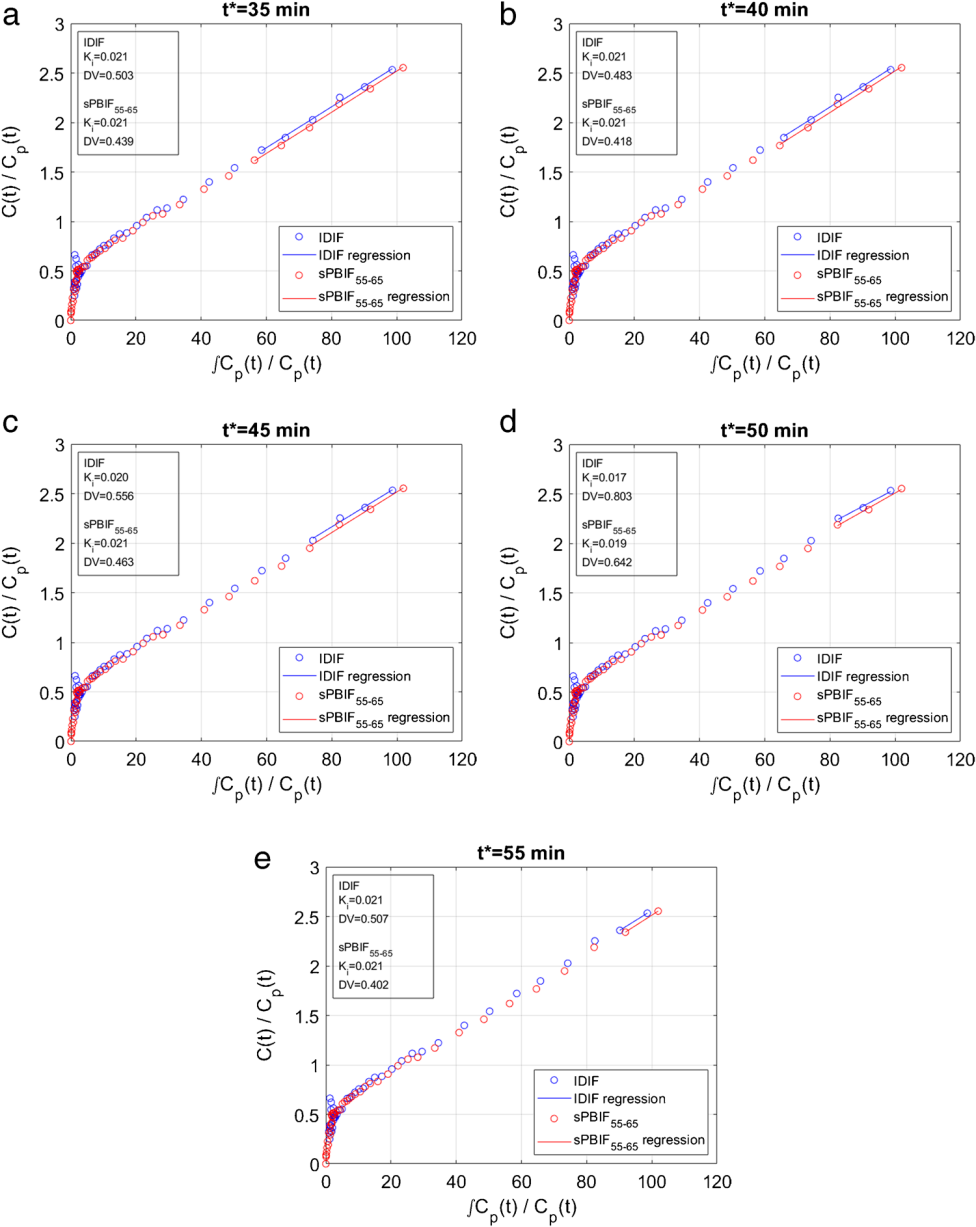
Patlak fits (solid lines) to ^18^F-FDG time activity curve from a lymphoma tumor lesion using IDIF (blue) and sPBIF_55–65_ (red). Patlak fits are shown for t* = 35 min (**a**), t* = 40 min (**b**), t* = 45 min (**c**), t* = 50 min (**d**), and t* = 55 min (**e**). The estimated Patlak parameters are shown in the inset boxes

**Fig. 4:**
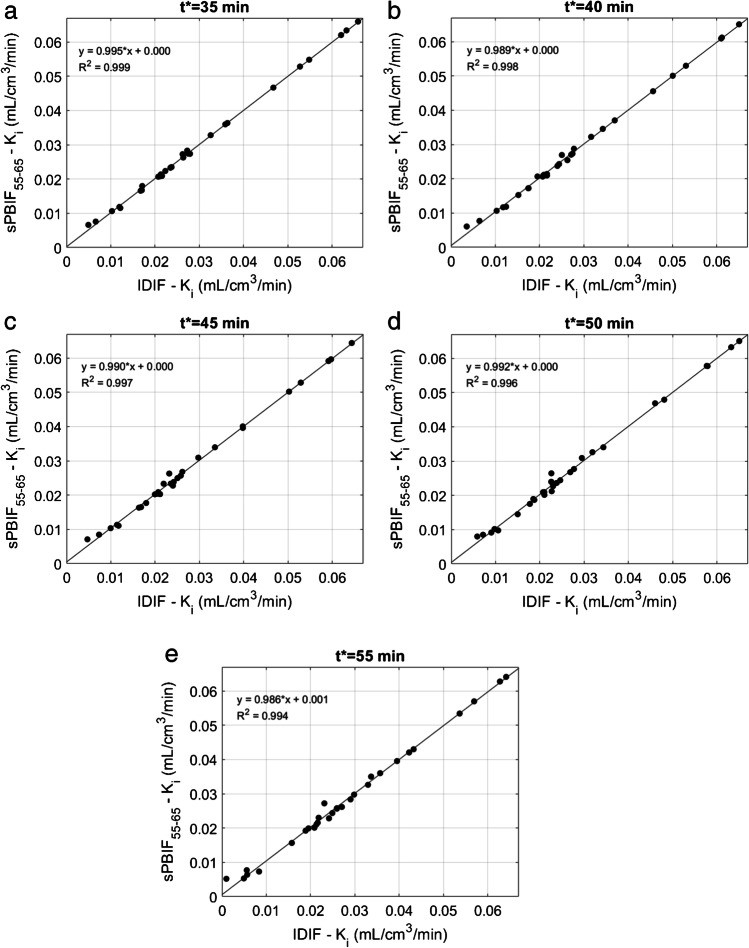
Graphs illustrating linear regression (solid lines) between Patlak *K*_i_ values from tumor lesions (*n* = 34) estimated using IDIF and sPBIF_55–65_ for t* = 35 min (**a**), t* = 40 min (**b**), t* = 45 min (**c**), t* = 50 min (**d**), and t* = 55 min (**e**). Very strong agreement between *K*_i_ values (*R*^2^ > 0.99) was observed at each t*

Examples of Patlak plots from a lymphoma tumor, including fits with IDIF and sPBIF_55–65_, are shown in Fig. 3 for different Patlak start time values. Similar curve shapes and Patlak slope (*K*_i_) estimates were obtained using IDIF and sPBIF_55–65_ at each of the t* values. As illustrated in Fig. 4, comparison of Patlak *K*_i_ values in 34 tumor lesions shows excellent agreement between IDIF and sPBIF_55–65_ estimates with *R*^2^ > 0.99. This correlation was present when different Patlak start times were used.

**Fig. 5:**
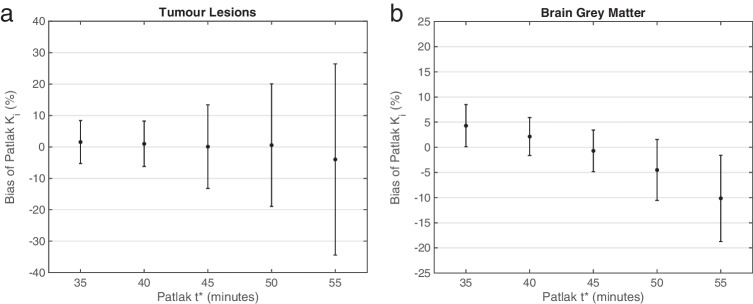
Comparison of percentage bias and standard deviation of bias (error bars) of *K*_i_ estimates using sPBIF_55–65_ with different Patlak start times (t*), compared against estimates with individual IDIF and standard t* of 35 min. Results are shown for tumor lesions (top) and brain gray matter (bottom)

Figure 5 illustrates the bias and precision of Patlak *K*_i_ for tumor lesions and brain gray matter estimated using sPBIF_55–65_ with varying Patlak start time, t*, calculated against reference *K*_i_ values estimated using IDIF and t* of 35 min. These results show that 20 min of PET data (45 to 65 min post-injection) is needed to achieve less than 1% bias and 15% precision error in tumor *K*_i_ estimates. In order to reduce the precision of error to less than 10%, 25 min of PET data was required. Linear regression *R*^2^ values were 0.99 for t* = 40 min and 0.98 for t* = 45. For brain gray matter, only 15 min of PET data (50 to 65 min post-injection) achieved less than 5% bias and precision error. Linear regression *R*^2^ values were 0.99 for t* = 45 and 0.94 for t* = 50 min.

**Fig. 6:**
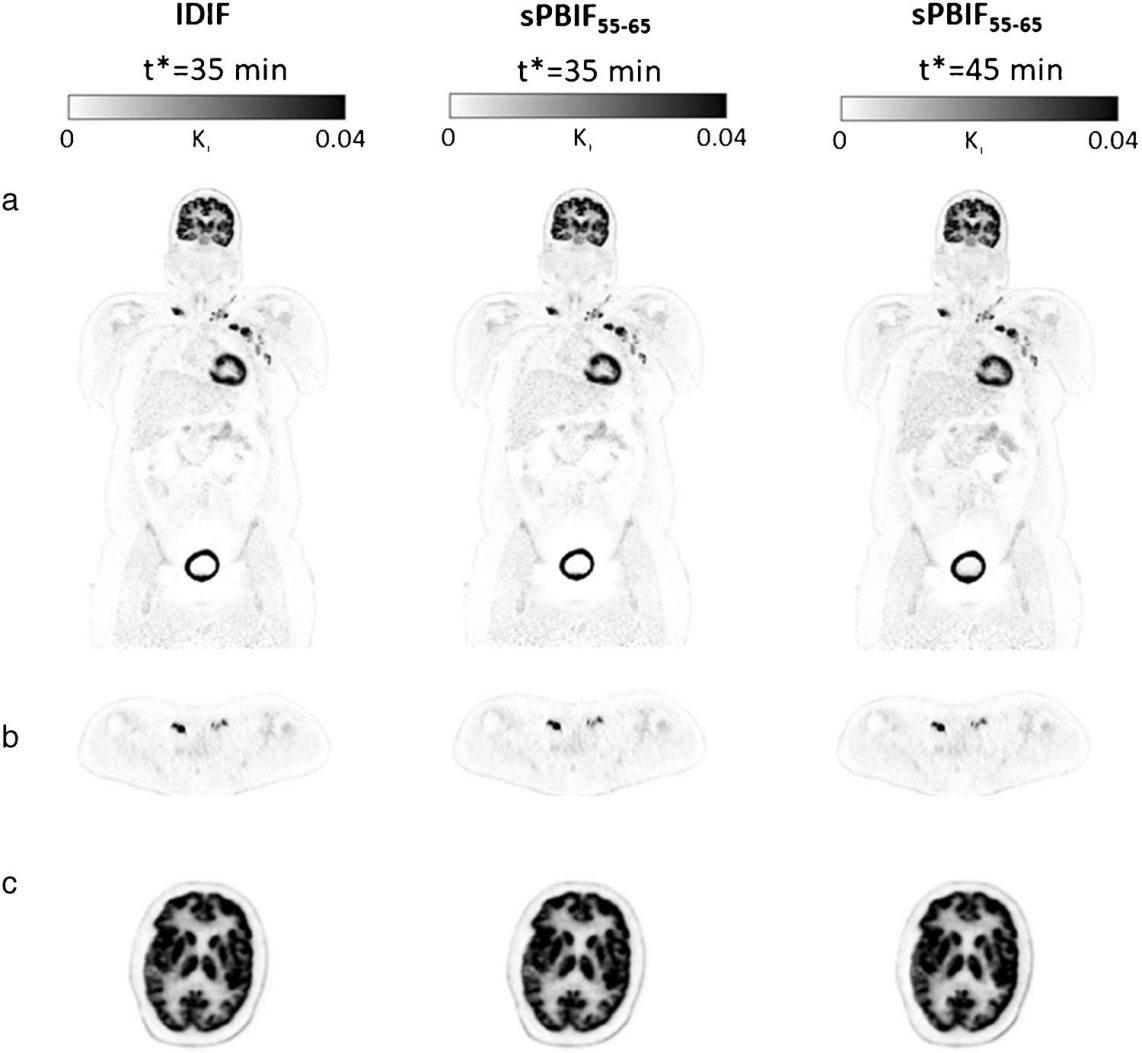
Whole-body Patlak *K*_i_ images were generated using 30 min of dynamic PET data (t* = 35 min) with IDIF, sPBIF_55–65_ and 20 min of PET data (t* = 45 min) with sPBIF_55–65_ for a representative lymphoma patient. (**a**) shows a coronal slice illustrating whole-body parametric images, (**b**) shows an axial slice containing reported lesions, and (**c**) shows an axial slice showing the brain

Figure 6 shows whole-body Patlak *K*_i_ images generated using IDIF and sPBIF_55–65_ with 30 min of PET data (t* = 35 min) and sPBIF_55–65_ with 20 min of PET data (t* = 45 min) for a representative subject with lymphatic cancer. The coronal slices illustrate the high qualitative resemblance of whole-body parametric images obtained using IDIF and sPBIF_55–65_ with 30 min of PET data (35 to 65 min post-injection) and sPBIF_55–65_ with 20 min of PET data (45 to 65 min post-injection). The axial slices shown in Fig. 6b and c illustrate the similar contrast between tumor and background regions and, likewise, between brain gray and white matter using both input functions. Computed over whole-body images, the average absolute relative error between Patlak *K*_i_ images generated using sPBIF_55–65_ with 30 min of PET data, compared against IDIF was 0.45 ± 0.29%. The absolute relative error increased to 1.00 ± 0.22% when sPBIF_55–65_ was used with 20 min of PET data (t* = 45). Parametric images generated using sPBIF_55–65_ with 20 min of PET data provided excellent image quality with SSIM > 0.99 and PSNR > 55 dB (Table 2).

**Table 2:** Non-absolute and absolute percentage relative change (RC), structural similarity index measure (SSIM), and peak signal-to-noise ratio (PSNR) of whole-body [^18^F]-FDG Patlak *K*_i_ images generated using sPBIF_55–65_ with PET data from 35 to 65 min post-injection (t* = 35 min) and 45–65 min p.i (t* = 45 min)

	Relative change (%)	Absolute relative change (%)	SSIM	PSNR (dB)
sPBIF_55–65_, t* = 35 min	0.31 ± 0.25	0.45 ± 0.29	0.998 ± 0.001	64.03 ± 3.59
sPBIF_55–65_, t* = 45 min	0.09 ± 0.03	1.00 ± 0.22	0.996 ± 0.002	55.06 ± 3.48

Patlak *K*_i_ images generated using IDIF and t* = 35 min served as reference

## Discussion

In this work, we have studied the use of population-based input functions with abbreviated dynamic [^18^F]-FDG protocols in a LAFOV PET system. Using 65-min long dynamic datasets obtained in oncological subjects undergoing scanning on a LAFOV Biograph Vision Quadra, we explored the optimal timing period to accurately scale the PBIFs using limited PET image data. We also investigated the feasibility of obtaining stable *K*_i_ estimates by using different Patlak linearization start times (t*) and, by analogy, using shorter examination protocols.

Although there is a substantial body of literature acquired over some four decades, kinetic modeling has yet to find an established role in routine oncological PET/CT imaging. One hindrance to its implementation is the requirement to scan for up to an hour, with application of the radiopharmaceutical on the scanning table. The advent of clinical LAFOV PET scanners with high sensitivity and time-of-flight resolution has brought renewed interest in this important methodology. In addition to enabling faster acquisition times and higher temporal sampling abilities in dynamic studies, LAFOV scanners can capture the entire body in a single FOV. This means that large blood pools or vascular structures, such as the aorta or left ventricle, can be exploited to yield an IDIF. In this present study, we derived individual IDIFs from the descending aorta and used these IDIFs to generate a PBIF. Kinetic analysis results with different sPBIFs show that scaling the PBIFs with an image-derived scaling factor served to estimate *K*_i_ with low bias (< 5%). This procedure eliminates the need for late arterial or venous samples, which are often required for PBIF scaling. Our results show that scaling the PBIFs with a scaling factor derived from 10 min of PET data (55–65 min post-injection) resulted in the lowest bias in tumor lesions and likewise in brain gray matter, indicating that this brief interval of PET data suffices for generating a reliable sPBIF. However, analysis of different Patlak linearization start times (t*) showed low precision (> 30%) but acceptable bias (− 4%) in tumor lesions when the fits were performed with 10 min of data (55–65 min p.i). Increasing the scan duration to 20 min (45–65 min p.i) improved the precision error to 13%, whereas 25 min of data (40–65 min p.i) resulted in 7% precision error in the estimated *K*_i_ values in tumor lesions. The [^18^F]-FDG data acquired within these time windows can also be used to generate a static image for more traditional SUV reading.

We have recently explored the fitness of abbreviated dynamic imaging protocols for LAFOV PET imaging with [^18^F]-FDG [15]. In that study, we found that two phase imaging protocol with dynamic PET data from 0 to 10–15 min post-injection followed by a 5-min scan at 60 min post-injection serves to estimate the magnitude of tumor *K*_i_ reliably (< 10% bias) [15]. In a similar work, Wu et al. showed that a dual imaging protocol that used PET data from 0 to 4 min and 54 to 60 min p.i can be used to estimate tumor *K*_i_ with 12–30% bias [14]. In that same study, Wu et al. also showed that a dual injection protocol can be used to estimate tumor *K*_i_ with a 10-min PET acquisition [14]. Use of PBIFs has also been evaluated with PET data acquired from SAFOV PET scanners. Results from Naganawa et al. showed that accurate *K*_i_ estimates with a precision error of 8–9% can be achieved by scaling the PBIF with image data from 30 to 60 min post-injection and then performing Patlak fitting to data from 60 to 90 min post-injection [21]. However, the required total scan duration with this protocol would still be 1 h, requiring substantial scanner time to realize which may hinder their routine implementation outside of research settings. In a similar work, van Sluis et al. showed that 30 min of PET data (30 to 60 min post-injection) might be adequate for accurate Patlak analysis of tumor lesions with PBIFs using simulated PET data [26]. In this study, we are able to demonstrate that *K*_i_ estimates with low bias and good precision are feasible using PBIF and 20-min clinical scans using a LAFOV system without recourse to additional blood sampling or dual-time point imaging protocols. Such an abbreviated protocol can be realized within time frames comparable to a routine clinical scan using established short-axial FOV systems [27]. These shortened protocols may pave the way for implementation of parametric imaging of PET data as part of clinical routine.

## Conclusion

Present results show that abbreviated protocols with a PBIF can serve for accurate Patlak linear graphic analysis of [^18^F]-FDG datasets from a LAFOV PET scanner. We demonstrate that 20 min of PET data (45–65 min post-injection) suffices for accurate kinetic modeling of tumor lesions, versus only 15 min (50–65 min post-injection) for brain gray matter. These abbreviated protocols exploiting a PBIF should enable wider implementation of quantitative parametric imaging protocols in a busy clinical setting.

## Supplementary Information

Below is the link to the electronic supplementary material.

Supplementary file1 (DOCX 108 KB)
