# Supplementary material for: Feasibility of using abbreviated scan protocols with population-based input functions for accurate kinetic modeling of [18F]-FDG datasets from a long axial FOV PET scanner
Source: Eur J Nucl Med Mol Imaging. 2022 Oct 4;50(2):257–65. doi: 10.1007/s00259-022-05983-7 (PMC9816288; doi:10.1007/s00259-022-05983-7)
Supplement: Supplementary file 1 — Supplementary file1 (DOCX 108 KB) [file 259_2022_5983_MOESM1_ESM.docx]

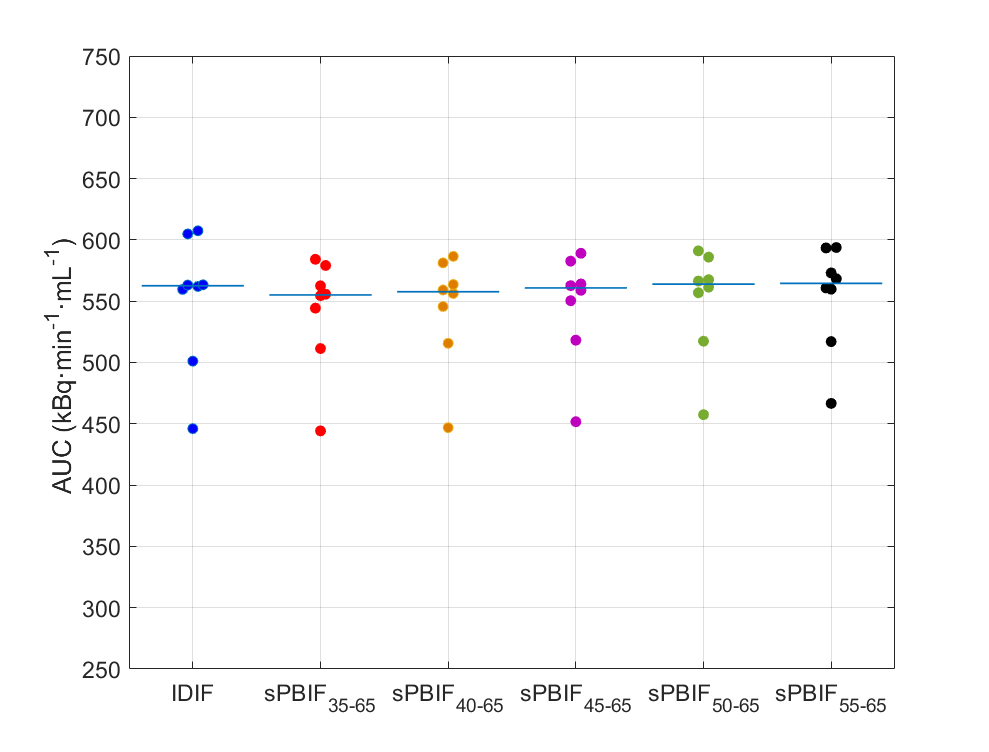


Supplementary figure 1: Dot plot of area under curves (AUCs) of IDIF and different sPBIFs with various scaling periods. Dots represent individual AUC values where the horizontal lines illustrate median AUC across each group.
